# Role of the Combination of Cyclin-Dependent Kinase Inhibitors (CDKI) and Radiotherapy (RT) in the Treatment of Metastatic Breast Cancer (MBC): Advantages and Risks in Clinical Practice

**DOI:** 10.3389/fonc.2021.643155

**Published:** 2021-06-17

**Authors:** Ambrogio Gagliano, Angela Prestifilippo, Ornella Cantale, Gianluca Ferini, Giacomo Fisichella, Paolo Fontana, Dorotea Sciacca, Dario Giuffrida

**Affiliations:** Department of Medical Oncology, The Mediterranean Institute of Oncology, Viagrande, Italy

**Keywords:** metastatic breast cancer (mbc), cyclin-dependent kinase inhibitors (CDKi), palbociclib, radiotherapy, toxicity, ribociclib, abemaciclib

## Abstract

Targeting cell cycle has become the gold standard for metastatic breast cancer (MBC), being cyclin-dependent kinase inhibitors (CDKIs) cornerstones of its treatment, alongside radiotherapy (RT). To date, no definite evidence regarding safety and efficacy of the combination of CDKIs plus radiotherapy (RT) is currently available. Purpose of this review is to collect data in favor or against the feasibility of the association of CDKIs + RT, describing its potential adverse events. Our review shows how CDKI + RT allows an overall satisfying disease control, proving to be effective and causing a grade of toxicity mainly influenced by the site of irradiation, leaning to favourable outcomes for sites as liver, spine or brain and to poorer outcomes for thoracic lesions or sites close to viscera; controversial evidence is instead for bone treatment. Toxicity also varies from patient to patient. To sum up, our contribution enriches and enlightens a still indefinite field regarding the feasibility of CDKIs + RT, giving cues for innovative clinical management of hormone-responsive MBC.

## Introduction

Up-to-date therapy of hormone-responsive metastatic breast cancer (MBC) is mainly based on cyclin-dependent kinase inhibitors (CDKI), nowadays considered cornerstones of its treatment. Examples of such agents are palbociclib, ribociclib and abemaciclib. These drugs CDKIs have been approved through three pivotal trials, namely PALOMA, MONALEESA and MONARCH for palbociclib, ribociclib, and abemaciclib, respectively. The main characteristics of such approving studies are summarized in **Supplementary Table 1** (1, 2), **Supplementary Table 2** (3, 4) and **Supplementary Table 3** (5, 6). CDKIs mechanism of action consists of the blockage of cyclin-dependent kinases 4 and 6, allowing the activation of RB oncosuppressor, thus halting tumor cell cycle in phase G1; main features regarding targets, use and toxicities recorded in a pivotal trial of theirs, are shown in **Supplementary Table 4** (1–3, 5, 7–15).

CDKIs, as **Supplementary Tables** show, are associated to endocrine therapy (ET) as best standard therapy for MBC HR+/HER2−, without visceral crisis or substantial organ impairment. Indeed, ET + CDKI provides better survival than ChT, with better RR than ChT, lesser toxicities and higher quality of life. Aromatase Inhibitors (AI) are currently preferred to tamoxifen as first line therapy in post-menopausal women, showing better TTP/PFS. OS was not proved to be higher than tamoxifen though. Recently, fulvestrant resulted more efficient than AIs as first line therapy in patients who did not receive any line of therapy. The combination of AI + CDKI showed higher efficacy than AI alone for hormone-sensitive patients. As second line therapy, for those who already received AI, CDKI + fulvestrant combination therapy showed better results than ET alone (16).

All the above given, no definite evidence regarding safety and efficacy of the combination of CDKIs plus radiotherapy (RT) is currently available. For instance, PALOMA trial encountered in the first place the issue of combining palliative RT with CDKIs: it was here indicated to suspend palbociclib from the day prior to RT to the seventh day following RT. Our purpose is to review available literature to collect data in favor or against the feasibility of the combination of CDKIs + RT, evaluating its efficacy and describing its potential toxicity.

## Methods

This review is based on clinical records collected across several cancer centers with scope of assessing possible advantages or disadvantages of CDKI + RT combination therapy. To select the relevant papers for the analysis, we performed a literature search on PubMed, updated until year 2020, with the following keywords: “RT + Palbociclib + metastatic breast cancer”; “RT + CDKI + metastatic breast cancer”. Overall, two letters to the editor, one review, five retrospective analyses and three case reports were selected and reviewed.

## Results

In all PALOMA studies patients who had bone lesions at the time of their enrolment benefited from palliative RT to improve pain, stopping palbociclib from the day prior to RT to the seventh day following RT. Patients who received such treatment were one in PALOMA 1, 16 in PALOMA 2 and nine in PALOMA 3 trials. Following the above-mentioned scheme, the time windows of the two treatments did not overlap ever, so no data were reported describing the interaction of Palbociclib + RT. Toxicities reported in literature hereby reviewed, are listed in **Table 1** below.

**Table 1:** Main studies regarding CDKIs + RT interaction.

Reference study design	No. of patients	Combination therapy	RT dose fraction and target	Type of toxicity	G1	G2	G3	G4
Hans et al., Radiother. Oncol (17).; Letter to the editor	5	Palbociclib + fulvestrant + RT	four backbone: **20 Gy/5**	Mucositis	1	1		
			one liver: **60 Gy/10**	Neutropenia			2	
				Anemia			1	
				Thrombocytopenia			2	
Kalash et al., Int J Radiat Oncol Biol Phys (18); Review	3	Palbociclib + letrozole + RT	two lung (dose missing)	two Irradiation pneumonitis				
			one liver (dose missing)	three Pulmonary fibrosis				
Kawamoto et al., Radiother. Oncol (19).; Letter to the editor	1	Palbociclib + fulvestrant + RT	Left iliac bone	Diarrhea (during RT)	1			
			First sacral vertebra	Bloody diarrhea, bloating, abdominal pain (after RT)				
			**30 Gy/10**					
Figura et al., J. Neurooncol (20).; Retrospective analysis	15	10 Palbociclib + RT	42 Brain mets	**-**				
		5 Abemaciclib + RT	**Median dose: 21 Gy (18–30 Gy)**					
			5: **18 Gy/1**					
			9: **20 Gy/1**					
			8: **21 Gy/1**					
			4: **24 Gy/1**					
			3: **20 Gy/5**					
			8: **25 Gy/5**					
			5: **30 Gy/5**					
Ippolito et al., Breast (21); Retrospective analysis	16	13 Palbociclib + RT	68.7% patients have bone mets	Neutropenia		2	4	1
		3 Ribociclib + RT	**(median dose: 30 Gy [8–36])**					
			31.2% patients have other site mets					
			**(median dose: 50 Gy [39.6–60])**					
Messer et al., Reports Pract. Oncol. Radiother (22).; Case report	1	Palbociclib + fulvestrant + RT	Supraclavicular metastatic node	Esophagitis				
			**(60 Gy/30)**	Dermatitis				
Chowdhary et al., Adv. Radiat. Oncol (23).; Retrospective analysis	16	Palbociclib + letrozole (10)/fulvestrant (6) + RT	11 Back bone	Leucopenia	8	1		
			four Pelvis					
			three Extremities					
			(16/18 bone mets: **30–37.5 Gy/10–15**; 1/18 bone mets: **18 Gy/1**;					
			1/18 bone mets: **30 Gy/3**)	Neutropenia	5	1		
			four Brain					
			**(30–35 Gy/10–14** for WBRT; **25 Gy/5** for fSRS)					
			Mediastinum: **36 Gy/18 fractions**	Thrombocytopenia	4			
Guerini et al., Sci. Rep (24).; Retrospective analysis	18	9 Palbociclib + RT	32 bone mets:	Ileitis			1	
		6 Ribociclib + RT	13/32: **30 Gy/10**					
		3 Abemaciclib + RT + letrozole	12/32: **20 Gy/5**					
		(8/18)/fulvestrant (10/18)	5/32: **8 Gy/1**					
			2/32: **30 Gy/3**					
				Neutropenia			11	
Ratosa et al., Clin. Breast Cancer (25); Retrospective analysis	46	30 Palbociclib + RT	50 Bone mets	–				
		15 Ribociclib + RT	seven Visceral mets					
		1 Abemaciclib + RT	three Brain mets					
			two Primary breast tumours					
			**(median dose: 20 Gy [8–63]/5 [1–28])**					
Dasgupta et al., J. Med. Radiat. Sci (26).; Case report	1	Palbociclib + letrozole + RT	Left pelvis and femur	Pancolitis				
			**30 Gy/10**					
Nasir et al., Anticancer Res (27).; Case report	1	Palbociclib + letrozole + RT	10th thoracic vertebra (T10): **2 Gy/5**	Esophagitis				
			Right iliac crest: **2 Gy/5**					
Meattini et al., The Breast (28)	5	Ribociclib + letrozole +	Bone (8) and visceral (2 lung, 1 liver) mets	Neutropenia				1
		RT	five bone mets: 4/5: 20 Gy/5 1/5: 30 Gy/5	Diarrhea + Vomiting				1
**Total No.**			**RT localization**	**Type of toxicity**	**G1**	**G2**	**G3**	**G4**
128			**Total 199**	Mucositis 2	1	1		
			Bone mets 101	Neutropenia 26	5	3	18	
			Liver mets 2	Anemia 1			1	
			Lung mets 4	Leucopenia 9	8	1		
			Brain mets 49	Thrombocytopenia 6	4		2	
			Primary breast tumors 2	Diarrhea 1				
			Other (unknown site) 41	Irradiation pneumonitis 2				
				Pulmonary fibrosis 3				
				Esophagitis 2				
				Dermatitis 1				
				Ileitis 1			1	
				

CDKI, Cycline-Dependent Kinase Inhibitor; Gy, Gray; MBC, Metastatic Breast Cancer; Mets, Metastases; No., Number; RTm Radiotherapy. Bold values regard radiotherapy and display erogated dose fraction and median dose fraction.

The analysis conducted by Hans et al. (17) explored the interaction of palbociclib + fulvestrant + RT in five patients with MBC, whose median age was 57.2. RT was here used to control pain and compression symptoms. Four patients received a dose of 20 Gy in five fractions to treat bone metastases, one patient underwent radiosurgery, receiving a dose of 60 Gy in 10 fractions for the treatment of liver metastases. All five patients obtained a symptoms control, and no significant skin toxicity was reported. Two patients developed grade 1/2 mucositis, two developed grade 3 neutropenia, one grade 3 anemia, and two grade 3 thrombocytopenia.

Another study was conducted by Kalash et al. (18) on three patients with MBC who received palbociclib + letrozole + RT. Two patients received RT on lung and one on chest wall. All three patients developed a severe pulmonary fibrosis. In addition, patients who received RT on lung developed a severe corticosteroid-resistant radiation pneumonia. Noteworthy, toxicity remitted after palbociclib suspension.

Kawamoto et al. (19) published a case report of a 58-year old woman with MBC and bone lesions who received palbociclib + RT combination therapy. The patient received palbociclib 100 mg every day for three weeks with one week stop and fulvestrant 500 mg every 14 days for the first three administrations, then every 4 weeks; subsequently, she received palbociclib and RT (30 Gy in 10 fractions) addressed to treat metastases on the iliac bone and on the first sacral vertebra. Reported adverse events were an episode of grade 1 diarrhea, left abdominal pain, swelling and bloody stool 3 days after last RT fraction. A CT scan and colonoscopy allowed to identify a radio-induced enterocolitis, responsive to a 3-week conservative management.

A further study addressed at evaluating the effects of CDKI + RT was conducted by Figura et al. (20) in patients with hormone-responsive MBC and brain metastases (BMs). The primary end point was brain toxicity during or after stereotactic RT, while control over BMs and overall survival (OS) were secondary end points. The final sample consisted of 15 patients, 10 treated with palbociclib and five with abemaciclib. Overall, 42 BMs were treated, 18 concurrently with CDKI, nine before and 15 afterwards, with a 9-month follow-up after stereotactic RT. Results showed that the combination of CDKI + cranial RT was well tolerated and effective in controlling BMs, displaying a survival benefit compared to conventional therapies.

In a study conducted by Ippolito et al. (21), a sample of 16 patients with hormone-responsive MBC was examined, 13 treated with palbociclib and three with ribociclib. RT treatment was administered to all of them, mainly with scope of palliation to bone disease, exception made for five patients who received RT at higher doses for local control of the lesion. Toxicities were mainly hematological, with neutropenia occurring the most: 12.5% of patients developed grade 2, 25% grade 3, and 6.3% grade 4 neutropenia. This study showed that the combination therapy seems to be safe, since adverse events consequent to either CDKI + RT or CDKI monotherapy were similar. Furthermore, it is of paramount importance to keep track of each patient’s history of adverse events, so to approach them appropriately in case of recurrence. In conclusion, since toxicities were mostly handled successfully, treatment was not modified.

Messer et al. (22), presented a case report of a woman with MBC who received RT on a metastatic supraclavicular lymph node with a total dose of 60 Gy in 30 fractions during palbociclib treatment. The patient reported early and severe side effects, such as esophagitis and dermatitis, heavily harming the left neck and requiring hospitalization. Therefore, palbociclib was suspended and RT completed, obtaining control over the neoplastic node. This study strongly underpins the importance of patients’ assessment prior to palbociclib + RT combination therapy, with timely treatment suspension where needed.

6A retrospective study conducted by Chowdhary et al. (23) collected cases ranging from 2015 to 2018 and evaluated the efficacy and toxicity of palbociclib + RT combination therapy. Overall, 16 patients were examined, four received palbociclib before RT, five concurrently and seven afterwards, for bone lesions (11 axial skeleton, four pelvis, three limbs), BMs (three whole brain RT and one stereotactic radiosurgery), and mediastinal lesions, respectively. It succeeded in relieving pain in every patient and detected adverse events were mainly haematological. Authors reported four cases of leukopenia, five of neutropenia and one of thrombocytopenia prior to RT, and five cases of leukopenia, one of neutropenia and three of thrombocytopenia after RT. Two grade 2 cases aside, all haematological toxicities were mainly grade 1. No grade 2 dermatological, neurological or gastrointestinal toxicities were notified, either in acute or afterwards. No differences were found based on either the timing of palbociclib + RT administration, or the number and anatomical site of irradiated lesions.

A recent retrospective study published by Guerini et al. (24) analyzed a group of patients with MBC treated with CDKI + RT. Toxicities were measured according to the Common Terminology Criteria for Adverse Events (CTCAE) 4.0, whilst local response was measured according to RECIST 1.1 or PERCIST 1.0, and pain control using a verbal numerical scale. Enrolled patients were 18, with 32 treated sites: they received palbociclib (50%) ribociclib (33.3%) and abemaciclib (16.7%). Acute non-haematological toxicity was not significant, exception made for a grade 3 ileitis. During the third month following RT, 61.1% of patients developed grade 3/4 neutropenia; however, no patient required permanent discontinuation of treatment. Pain control was fully achieved in 88.2% of patients three months after RT; 94.4% of patients obtained and maintained local disease control.

Another retrospective study was recently published by Ratosa et al. (25). On a sample of 46 patients with MBC treated with CDKI + RT, 30 receiving palbociclib, 15 ribociclib and one abemaciclib, with a total number of 62 sites treated with RT, for 50 bone lesions, seven visceral metastases, three BMs and two primary breast tumors. Overall, grade 3 or higher adverse events rates were 6.5% prior to RT, 4.3% during RT, 15.2% at 2nd week and 23.9% at 6th week after RT.

A case report presented by Dasgupta et al. (26) described a 77-year-old woman with hormone-dependent MBC treated with palbociclib and palliative RT on left pelvis and femur. Five days after RT, she developed pancolitis which required hospitalization.

Another case report was recently published by Nasir et al. (27) describing the visceral toxicity of CDKI + RT association. A 63-year-old patient receiving palbociclib and RT developed odynophagia and dysphagia due to the presence of grade 2/3 esophageal ulcers. Symptoms improved after discontinuation of the association, allowing the patient to continue palbociclib therapy.

In 2018, Meattini et al. (28) published preliminary data regarding five patients suffering from MBC treated with ribociclib plus letrozole and concomitant palliative RT as first-line treatment. Three patients had both bone and visceral disease and two patients had bone disease only. RT was never interrupted, and the palliative intent was achieved. Letrozole was also not suspended. Ribociclib was discontinued for two weeks in two cases: firstly, a G3-G4 neutropenia and secondly, a case of G3-G4 diarrhea and vomiting. At 3-month follow up, three stable disease and two partial response were observed, showing encouraging results regarding the combination of ribociclib, letrozole and RT.

In 2020, Meattini et al. (29) published a retrospective analysis, in form of abstract, of direct administration of RT to metastases in combination with first or second-line treatment with CDKIs for MBC.

The study involved 85 consecutive patients treated with CDKI between 2017 and 2019, 22 of these received ribociclib and 63 palbociclib. Overall, 29.4% of patients (n = 25) received metastases-directed RT during CDKI administration: specifically, 16.5% (n = 14) were treated concurrently, 12.9% (n = 11) subsequently.

The main endpoints of the analysis were the impact of RT on CDKI treatment, such as dose reduction or discontinuation, adverse events of any grade plus grade ≥2, and grade ≥2 neutropenia according to CTCAE (Common Terminology Criteria for Adverse Events) scale version 5.0.

Finally, they observed that metastases-directed RT did not cause dose reduction or discontinuation of CDKI, as proved by the stationary rate of adverse events: indeed, there was no difference in terms of CDKI dose reduction or treatment discontinuation, toxicity of any grade or grade ≥2, neutropenia grade ≥2 between patients receiving RT vs. no RT and between groups receiving concomitant RT vs. sequential RT vs. no RT.

## Discussion

As previously mentioned, PALOMA trials in the first place encountered the issue of combining palliative RT with CDKIs. In this instance, it was indicated to suspend palbociclib from the day prior to RT to the seventh day following RT, as authors recommend doing for other CDKI agents too. According to **Table 1**, it is possible to compare grade 1/2 toxicities to grade 3/4 toxicities; two mucositis all fall in grade 1/2 events; 26 neutropenia fall mainly instead in the grade 3/4 category (18), than in the grade 1/2 category (8); a reported anemia event was of grade 3; nine cases of leukopenia are all of grade 1/2; among six cases of thrombocytopenia, four were grade 1/2, two grade 3; the only reported ileitis was of grade 3; overall, among 45 overall cases of reported degree toxicities, 22 were of grade 3/4 (18 (82%) of them were all represented by neutropenia events), while 23 were of grade 1/2.

First off, an evidence emerging from this analysis concerns the not yet clarified correlation between anatomical site of treated lesions and toxicity degree induced by CDKI + RT. Evidence in support is provided by the study conducted by Hans et al., highlighting satisfying symptoms control alongside low-grade blood toxicity and mild mucositis in MBC patients treated with CDKI + RT for spine or liver metastases. Conversely, as backed by Kalash et al., MBC patients treated alike, though for chest wall or lung lesions, suffered from severe adverse events, such as pulmonary fibrosis and radiation pneumonia, ultimately determining treatment suspension. Thoraco-pulmonary lesions seem to show higher risk of more severe adverse events than bone or liver localization when treated with RT. However, it still is contradictory whether RT treatment on bone lesions spares the patient severe toxicities: as described by Kawamoto et al. in their case report, where irradiation regarded the iliac bone involving part of the bowel, it caused diarrhea and acute radiation-induced enteritis. Similar considerations apply to the case report published by Dasgupta et al. on a 77-year-old woman who developed a severe pancolitis following RT to pelvis and femur in combination with palbociclib. The authors warn to record patient’s gastrointestinal history and to adopt the best RT techniques to reduce the dose of radiation, thus minimizing any potential side effect.

Therefore, on one hand, RT seems safer on spine than on chest wall or lung; on the other hand, the site of targeted metastasis is crucially relevant: if it is close to viscera, such as bowels, side effects could be so severe that stopping the combination therapy might be mandatory. According to Figura et al, as far as palbociclib + RT on BMs is regarded, evidence shows that it is more effective in symptoms and disease control than palbociclib monotherapy, positively affecting the survival too and not causing any extra toxicity than palbociclib only. Increase in toxicity due to palbociclib + RT combination therapy was instead claimed by the case report of Messer et al., in which RT on superficial lesions determined esophagitis and acute dermatitis, requiring hospitalization. Same combination therapy was used in the case report of Nasir et al., which caused severe esophagitis with grade 2/3 ulcers, alongside odynophagia and dysphagia. In both cases treatments were suspended. From all the evidence above, it can be said that oncologists should be able to foresee and manage in advance any mucositis and dermatitis in case of administration of CDKI + RT combination therapy. The study conducted by Ippolito et al., underpins the relevance of assessing potential toxicities of palbociclib + RT association based on patient’s personal history. Indeed, adverse events are highly associated to pre-treatment conditions and individual features, in both this regimen and palbociclib alone. Therefore, it is highly recommended for oncologists to predict and prevent toxicity before even combining a CDKI with RT. Latest retrospective analyses seem to confirm the efficacy and safety of CDKI + RT. For instance, the study conducted by Guerini et al., shows that the main adverse event was neutropenia; moreover grade 3/4 neutropenia rate was comparable to the one detected with CDKI monotherapy. The most interesting aspect concerns pain improvement and disease local control. Data provided by the retrospective study conducted by Ratosa et al., highlighted the improvement of pain symptoms in an 80% of patients.

Finally, we found that several ongoing studies are focussed on proving the potential radiosensitizing effect of palbociclib, to make cancer cells more susceptible to RT. We here report the experimental study of a Spanish research team carrying out an analysis exploring palbociclib as a radiosensitizer on lung, colorectal and breast cancer cells (30). The results showed that wild type p53 is strictly needed for palbociclib to act as a radiosensitizer; oppositely, palbociclib loses any radiosensitizing efficacy when p53 is functionally blocked, reacquiring it when p53 is restored. These data provide cues for a more patient-tailored therapy, in which responders to CDKI + RT would only be those with a normally functioning p53 pathway (30).

## Conclusions

According to collected data, it can be concluded that the combination of CDKI + RT allows an overall satisfying disease control, proving to be effective and causing a grade of toxicity influenced by some factors as the site of irradiation, leaning to favourable outcomes for sites as liver, spine or brain and to poorer outcomes for thoracic lesions or sites close to viscera; controversial evidence is instead for bone treatment. Toxicity also varies from patient to patient: in this context, the acknowledgment of toxicity and comorbidities history becomes of crucial importance. Therefore, according to our analysis we believe that the association of CDKI + RT might be effective and safe, and it is surely deserving more deepening through further analyses.

## Author Contributions

Conception and design: AG and AP. Administrative support: OC. Provision of study materials: AG, AP, GFe, GFi, PF, DS, and DG. Collection and assembly of data: AG and AP. Data interpretation: AG and OC. All authors contributed to the article and approved the submitted version.

## Conflict of Interest

The authors declare that the research was conducted in the absence of any commercial or financial relationships that could be construed as a potential conflict of interest.
