# Supplementary material for: Role of the Combination of Cyclin-Dependent Kinase Inhibitors (CDKI) and Radiotherapy (RT) in the Treatment of Metastatic Breast Cancer (MBC): Advantages and Risks in Clinical Practice
Source: Front Oncol. 2021 Jun 17;11:643155. doi: 10.3389/fonc.2021.643155 (PMC8247460; doi:10.3389/fonc.2021.643155)
Supplement: Supplementary file 1 [file DataSheet_1.pdf]

Supplementary table 1: key approval studies for PALBOCICLIB

	Study design and Endpoints	Patients enrolled	Study arms	Results
**Paloma-2**	Multicenter, randomized 2:1, double-blind, placebo controlled, phase 3 trial<br><br>Primary Endpoint: Investigator-assessed PFS<br><br>Other: Investigator-assessed OR	**666** postmenopausal women with HR+/ HER2- BC who did not receive any prior systemic anti cancer treatment for advanced disease	**444** letrozole (2.5 mg once daily) + palbociclib (125 mg once daily for 21 days of every-28-day cycle, followed by 7 days off treatment);<br><br>**222** letrozole (2.5 mg once daily) + placebo (once daily for 21 days of every-28-day cycle, followed by 7 days off treatment)	**Investigator-assessed PFS**:<br>**27.6** mos. (palbociclib + letrozole)<br>**14.5** mos. (placebo + letrozole)<br><br>**Investigator-assessed OR**:<br>**47.5%** (palbociclib + letrozole)<br>**38.7%** (placebo + letrozole)
**Paloma-3**	Multicenter, randomized 2:1, double-blind, placebo-controlled phase 3 trial<br><br>Primary Endpoint: Investigator-assessed PFS<br><br>Other: OR	**521** women (any menopausal status) with HR+/HER2- ABC that relapsed or progressed during prior ET During study treatment, pre- and perimenopausal women must be receiving therapy with the LHRH agonist goserelin.	**347** palbociclib (125 mg per day for 3 weeks followed by 1 week off) ± fulvestrant (500 mg on days 1 and 15 of cycle 1 and then every 28 days). Pre- and perimenopausal women received goserelin;<br><br>**174** placebo (3 weeks followed by 1 week off) ± fulvestrant (500 mg on days 1 and 15 of cycle 1 and then every 28 days). Pre- and perimenopausal women received goserelin.	**Investigator-assessed PFS**:<br>**9.2** mos. (palbociclib + fulvestrant +/- goserelin)<br>**3.8** mos. (placebo + fulvestrant +/- goserelin)<br><br>**OR**:<br>**10.4%** (palbociclib + fulvestrant +/- goserelin)<br>**6.3%** (placebo + fulvestrant +/- goserelin)

Abbreviations: ABC: Advanced Breast Cancer; ET: Endocrine Therapy; HER2: Human Epidermal Growth Factor Receptor 2; ORR: Odds Ratio; PFS: Progression-Free Survival; HR: Hormone Receptor; NSAI: NonSteroidal Aromatase Inhibitor;

Supplementary table 2: key approval studies for RIBOCICLIB				
	**Study design and Endpoints**	**Patients enrolled**	**Study arms**	**Results**
**Monaleesa-2**	Multicenter, randomized 1:1, double-blinded, placebo-controlled trial<br><br>Primary Endpoint: PFS<br><br>Other: OS ORR	**668** postmenopausal women with HR+/HER2- ABC, who received no prior treatment for advanced disease	**334** ribociclib (600 mg/day; 3 weeks-on/1 week-off; 28-day treatment cycles) plus letrozole (2.5 mg/day; continuous);<br><br>**334** placebo plus letrozole.	PFS:<br>**25.3** mos. ribociclib + letrozole)<br>**16.0** mos. placebo + letrozole<br>OS:<br>Not data reported<br><br>ORR:<br>**40.7%** (ribociclib + letrozole)<br>**27.5%** (placebo + letrozole)
**Monaleesa-7**	Multicenter, randomized 1:1, double-blinded, placebo controlled trial<br><br>Primary Endpoint: PFS<br><br>Other: OS ORR	**672** premenopausal women with HR+/(HER2-) ABC	**335** ribociclib (600 mg/day; 3 weeks-on/1 week-off; 28-day treatment cycles) plus NSAI or tamoxifen (tamoxifen 20 mg daily oral or letrozole 2.5 mg daily oral or anastrozole 1 mg daily oral) and goserelin 3.6 mg subcutaneous injection (once every 28 days);<br><br>**337** placebo (600 mg daily oral (3 weeks on/ 1 week off) plus NSAI or tamoxifen (tamoxifen 20 mg daily oral or letrozole 2.5 mg daily oral or anastrozole 1 mg daily oral) and goserelin 3.6 mg subcutaneous injection (once every 28 days).	PFS:<br>**23.8** mos. (ribociclib + NSAI/tamoxifen + goserelin) versus **13.0** mos. (placebo + NSAI/tamoxifen + goserelin);<br><br>OS:<br>NE (ribociclib + NSAI/tamoxifen + goserelin);<br>**40.9** months (placebo + NSAI/ tamoxifen + goserelin)<br><br>ORR:<br>**40.9 %** (ribociclib + NSAI/tamoxifen + goserelin)<br>**29.7 %** (placebo + NSAI/tamoxifen + goserelin)

Abbreviations: ABC: Advanced Breast Cancer; HER2: Human Epidermal Growth Factor Receptor 2; ORR: Overall Response Rate; OS: Overall Survival; PFS: Progression-Free Survival; HR: Hormone Receptor; NSAI: NonSteroidal Aromatase Inhibitor;

Supplementary table 3: key approval studies for ABEMACICLIB

	Study design and Endpoints	Patients enrolled	Study arms	Results
**Monarch-2**	Multi-center, randomized 2:1, double-blinded, placebo controlled phase 3 trial<br><br>Primary Endpoint: PFS<br><br>Other: ORR	**669** women HR+/ HER2- locally advanced or MBC	**446** abemaciclib (150 mg twice daily in 28-day cycles) + fulvestrant 500 mg (250 mg on Day 1 and 250 mg on Day 15 of Cycle 1, then on Day 1 of Cycle 2 and beyond);<br>**223** placebo (150 mg twice daily in 28-day cycles) + fulvestrant 500 mg (250 mg on Day 1 and 250 mg on Day 15 of Cycle 1, then on Day 1 of Cycle 2 and beyond).	PFS:<br>**16.4** mos. (abemaciclib + fulvestrant)<br>**9.3** mos. (placebo + fulvestrant)<br><br>ORR:<br>**48.1%** (abemaciclib + fulvestrant)<br>**21.3%** (placebo + fulvestrant)
**Monarch-3**	Multi-center, randomized, double-blinded, phase 3, placebo controlled phase 3 trial<br><br>Primary Endpoint: PFS<br><br>Other: ORR	**493** postmenopausal women HR+/HER2- locally advanced or MBC with no prior systemic therapy in this disease setting	**328** abemaciclib (150 mg twice daily) + 1 mg anastrozole or 2.5 mg letrozole once daily (28 day cycles);<br>**165** placebo (150 mg twice daily) + 1 mg anastrozole or 2.5 mg letrozole once daily (28 day cycles).	PFS:<br>**28.18** mos. (abemaciclib + anastrozole/letrozole)<br>**14.76** mos. (placebo + anastrozole/letrozole)<br><br>ORR:<br>**49.7 %** (abemaciclib + anastrozole/letrozole)<br>**37.0%** (placebo + anastrozole/letrozole)

Abbreviations: ET: Endocrine Therapy; HER2: Human Epidermal Growth Factor Receptor 2; HR: Hormone Receptor; MBC: Metastatic Breast Cancer; NSAI: NonSteroidal Aromatase Inhibitor; ORR: Overall Response Rate; PFS: Progression-Free Survival;

Supplementary table 4: CDKIs key characteristics

	Palbociclib	Ribociclib	Abemaciclib
**Target**	CDK4; CDK6		
**Route**	Per Os		
**Indications**			
- *CDK4/6I + NSAI*[4]	Pre/perimenopausal women, with HR+/HER2− ABC; No prior ET for MBC; ≤ 1 prior ChT for ABC		
- *CDK4/6I + Fulvestrant*	Women any menopausal status, with HR+/HER2- ABC that relapsed or progressed during prior ET, ≤1 line of ChT for ABC	Postmenopausal women and men with HR+/HER2- ABC, 0-1 line of ET for ABC	Women any menopausal status, with HR+/HER2- ABC that had progressed during prior ET, ≤1 ET, no prior ChT for ABC
**Line of treatment:**			
- *First*	+ AI (letrozole)	+ AI (letrozole)<br>+ NSAI[4]	+ anastrozole/letrozole/fulvestrant
- Second	+ fulvestrant	+ fulvestrant	+ fulvestrant<br><br>*Monotherapy when PD on or after ET and 1-2 ChT regimens

	**Palbociclib + letrozole** (PALOMA-2, n=444)	**Ribociclib + letrozole** (MONALEESA-2, n=334)	**Abemaciclib + letrozole** (MONARCH-3, n=327)
**Toxicities (%):**			
- *Neutropenia*	79,5 (G3/4: 56,1/10,4)	76,9 (G3/4: 52,4/9,6)	41,3 (G3/G4: 19,6/1,5)
- *QTcF prolongation*	NR	3%	NR
- *Diarrhoea*	26,1 (G3/4: 1,4/0)	38,3 (G3/4: 2,4/0)	81,3 (G3/4: 9,5/0)
- *Vomiting*	15,5 (G3/4: 0,5/0)	33,5 (G3/4: 3,6/0)	28,4 (G3/4: 9,5/0)
- *Abdominal pain*	11,3 (G3/4: 0,9/0)	NR	29,1 (G3/4: 1,2/0)
- *Hyporexia*	14,9 (G3/4: 0,7/0)	20,7 (G3/4: 1,5/0)	24,5 (G3/4: 1,2/0)
- *Increased ALT*	43 (G3/4: 2/<1)	NR	15,6 (G3/4: 5,8/<1)
- *Increased AST*	52 (G3/4: 3/0)	NR	15 (G3/4: 3/0)
- *Fatigue*	37,4 (G3/4: 1,8/-)	41,3 (G3/4: 2,7/0,3)	40,1 (G3/4: 1,8/-)

Abbreviations: ABC: Advanced Breast Cancer; AI: Aromatase Inhibitor; AST: Aspartate Aminotransferase; ALT: Alanine Aminotransferase; CDK: Cyclin-Dipendent Kinase; CKDI: CDK Inhibitor; ChT: Chemotherapy; ET: Endocrine Therapy; HER: Human Epidermal Growth Factor Receptor; N: Number; NR: Not Reached; NSAI: Non-Steroideal Aromatase Inhibitor; PD: Progression Disease; QTcF: corrected QT interval by Fredericia;
